# Whether a Gluten-Free Diet Should Be Recommended in Chronic Autoimmune Thyroiditis or Not?—A 12-Month Follow-Up

**DOI:** 10.3390/jcm10153240

**Published:** 2021-07-22

**Authors:** Jakub Pobłocki, Tamara Pańka, Małgorzata Szczuko, Arkadiusz Telesiński, Anhelli Syrenicz

**Affiliations:** 1 Department of Endocrinology, Metabolic Diseases and Internal Diseases, Pomeranian Medical University, 70-252 Szczecin, Poland; tamara@panka.eu (T.P.); klinedno@pum.edu.pl (A.S.); 2 Department of Human Nutrition and Metabolomic, Pomeranian Medical University, 71-460 Szczecin, Poland; 3 Department of Bioengineering, Faculty of Environmental Management and Agriculture, West Pomeranian University of Technology, 71-434 Szczecin, Poland; arkadiusz.telesinski@zut.edu.pl

**Keywords:** cAITD, gluten free diet, celiac disease, Hashimoto’s thyroiditis, antithyroid antibodies

## Abstract

Elimination diets have recently become extremely popular among people with autoimmune diseases. A gluten-free diet is indicated in celiac disease (CD), but some studies show its effectiveness in cases of autoimmunity. The aim of this study was to assess whether the use of a gluten-free diet is also effective in patients with chronic autoimmune thyroid disease (cAITD), which is the most common thyroid autoimmune pathology associated with chronic inflammation, over-reactivity of the immune system, auto-destruction of thyrocytes and hypothyroidism. The final analysis of the study included 62 Caucasian women randomized into a control group (CG: *n* = 31) and an experimental group on a gluten-free diet (GFDG: *n* = 31), were subject to a 12-month follow-up, during which the concentrations of thyrotropin (TSH), free triiodothyronine (fT3), free thyroxine (fT4), anti-thyroid peroxidase (anti-TPO) and anti-thyroglobulin (anti-TG) antibodies were assessed at baseline and after 3, 6 and 12 months. During the 12-month follow-up between the CG and the GFDG, no differences were found in anti-TPO and anti-TG antibodies, fT3 or fT4 levels, except a significant reduction in TSH levels in the GFDG. Additionally, performed analysis between individual appointments presented no significant differences in changes in the median concentrations of anti-TPO, anti-TG or fT3, but confirmed a significant decrease in TSH and showed accessory an increase in fT4 after 12 months in GFDG. Statistical analyses performed separately for both groups indicated a constant reduction of anti-TG concentrations in the GFDG. In conclusion, a GFD may be administered in cAITD after ruling out celiac disease, but it is necessary to perform more studies to assess if cAITD patients achieve the benefits of following a GFD. Patients with cAITD should be offered proper nutrition education combined with a healthy lifestyle promotion.

## 1. Introduction

Chronic autoimmune thyroid disease (cAITD), i.e., Hashimoto’s thyroiditis (HT), is one of the most prevalent autoimmune thyroid pathologies. Diagnosed predominantly in women (female to male ratio = 12:1), it is the most common cause of primary hypothyroidism in developed, iodine-sufficient populations [1,2].

Thyrocyte destruction in HT is mostly a consequence of an active, chronic autoimmune process, involving CD^4+^ and CD^8+^ T lymphocytes, B CD^19+^ cells, macrophages and plasma cells [3,4,5].

There is evidence of increased prevalence of other endocrine and non-endocrine autoimmune diseases (AID), such as: type 1 diabetes, Graves’ disease, Addison’s disease, myasthenia gravis, Sjögren’s syndrome, rheumatoid arthritis, rheumatic polymyalgia, primary sclerosing cholangitis, chronic gastritis, vitiligo, psoriasis and psoriatic arthritis, alopecia areata, Addison–Biermer anemia and celiac disease (CD) in HT patients [6]. HT is therefore considered one of the autoimmune polyendocrine syndromes (APS) [6].

The diagnosis of HT is based on a combination of clinical features, the presence of serum anti-thyroid peroxidase (anti-TPO) and anti-thyroglobulin (anti-TG) antibodies and reduced echogenicity of the thyroid gland [1,2]. Continuous destruction of the thyroid parenchyma in HT leads to hypothyroidism, and patients with HT require chronic therapy with preparations of levothyroxine [2].

The prevalence of CD in patients with autoimmune thyroid diseases (AITD) is estimated to range between 2 and 7.8%, while in the population with cAITD it settles at about 4.8%. In turn, autoimmune thyroid disease in CD patients occurs with the frequency of 5.4–26.3% [7,8]. Although HT and CD seem to have a shared genetic background, it is known that the genetic disposition itself, without the contribution of environmental factors, does not automatically lead to the development of either one of the disorders. In fact, only 3% of the 40% of European carriers of the HLA-DQ2 and HLA-DQ8 alleles, which raise the risk of CD, do develop CD [9]. The agent responsible for the induction of the autoimmune process in CD, whose mechanism is quite well-known, is gluten, a protein found in durum, spelt and Khorasan wheat (gliadin), barley (hordein), rye (secalin) and triticale hybrids [10,11,12].

Positive tissue transglutaminase 2 (anti-tTG) antibodies, anti-endomysial antibodies (EMA) and antibodies for deamidated gliadin peptides (DGP) are characteristic markers for CD, and the gold standard for confirming the diagnosis in children, adolescents and adults is still the histopathological examination of the duodenal mucosa, which proves villous atrophy [13]. It is worth mentioning that since 2012 for children and adolescents, the biopsy, under specific circumstances, is not needed [14,15].

A gluten elimination diet in patients with CD is reported to inhibit the autoimmune process, reduce the concentration of anti-tTG in the blood serum and contribute to the withdrawal of pathological changes in the small intestine [16]. A gluten-free diet (GFD) is the only treatment for CD, wheat allergy, gluten ataxia, herpetic dermatitis (Duhring’s disease) and non-celiac gluten sensitivity [17,18,19].

According to some reports, a GFD can be beneficial in other autoimmune diseases such as rheumatoid arthritis, where it can have an anti-inflammatory effect and improve symptoms in case of resistance to conventional drug therapies [20].

A lifelong GFD in non-obese diabetic (NOD) mice reduces the infiltration of monocytes/macrophages and possibly T lymphocytes in the salivary glands and reduces the severity of pancreatic islet inflammation [21].

Despite the growing interest in GFDs among HT patients, there are currently no indications for a GFD in chronic autoimmune thyroiditis (cAITD). Therefore, we have decided to investigate whether administration of a GFD in HT can lead to an improvement in terms of immunological and hormonal parameters.

## 2. Materials and Methods

### 2.1. Study Sample

The sample of our prospective study included a total of 92 Caucasian women aged 18–55 with diagnosed cAITD, whose diets consisted of at least one meal a day containing approx. 10 g of gluten, i.e., about 4 slices of bread [22], for a period of at least 3 months prior to enrollment. HT was diagnosed based on the ultrasound image typical of cAITD (ALOKA Prosound Alpha-7 apparatus, UST-5411 4.4–13.3 MHz linear probe, Japan) and elevated blood serum levels of anti-TPO (>34 IU/mL) and/or anti-TG (>115 IU/mL) [23]. Thyroid hormone status was defined by measurement of: TSH (0.270–4.200 µIU/mL), fT4 (0.93–1, 70 ng/dL) and fT3 (2.00–4.40 pg/mL). Exclusion criteria were: a gluten-free diet or periodic elimination of gluten from the diet, malabsorption syndromes, bariatric surgery, intestine or stomach resection, removal of the thyroid gland, treatment with 131-I, Graves’ disease or its clinical symptoms, diabetes, hypertension, coronary artery disease, active inflammation, use of glucocorticosteroids, statins, non-steroidal anti-inflammatory drugs and immunosuppressants or drugs affecting the thyroid axis other than levothyroxine and smoking. Upon receiving comprehensive information on the course of the study, all patients provided consent to participate. cAITD diagnosis was confirmed in all patients, then the concentration of IgA-class antibodies against tTG and total IgA were determined in order to test for CD. Due to the elevated concentrations of anti-tTG, two patients underwent gastroscopy and duodenal biopsy. No evidence of villous atrophy in the histopathological examination was allowed to rule out CD. Due to hypothyroidism, 83 patients received levothyroxine replacement therapy.

### 2.2. Group Selection

A total of 92 study participants with cAITD were randomly assigned to the control (CG; *n* = 50) and gluten-free diet group (GFDG; *n* = 42) and subjected to a 12-month observation. Serum concentrations of TSH, fT3, fT4, anti-TPO and anti-TG antibodies were assessed at baseline and after 3, 6 and 12 months. Due to failure to report for follow-up within the prescribed period, the sizes of both groups varied throughout the 12-month observation (at 3 months CG (*n* = 35) and GFDG (*n* = 35), at 6 months CG (*n* = 32) and GFDG (*n* = 28) and at 12 months CG (*n* = 31) and GFDG (*n* = 31)). Ultimately, the statistical analysis included patients who attended at least 3 appointments, and thus the control and study groups were *n* = 31 and *n* = 31, respectively. All 62 women were receiving levothyroxine because of hypothyroidism or subclinical hypothyroidism, and were clinically euthyroid.

The reasons for dropping out from the GFD group included: pregnancy (*n* = 2), difficulties in following a GFD (*n* = 3) and failure to adhere to the recommendations regarding appropriate and compliant substitution of levothyroxine (*n* = 2), while in the control group, those were: pregnancy (*n* = 6) and endometriosis (*n* = 1). All remaining patients failed to provide the reasons for dropping out from the study.

### 2.3. Control Group

The control group was recruited randomly from women with Hashimoto’s disease at the same time as the women in the study group. Thus, the impact of seasonal product differentiation was limited. There was general information about the study and its objectives advertised via social media and paper flyers. The willingness of the patient to join the participation was reported by phone. During the telephone calls, the following criteria were valued: no changes in dietary habits in 3 months prior to the study, including the use of elimination diets. The diet of the control patients did not undergo any modification; they consumed gluten before and during the study. Overall, 50 women consented to take part in the study, but to the control visit, 35 (3 months), 32 (6 months) and 31 (12 months) patients attended, respectively. The control group consisted of 31 women, aged 37.07 (33.83–40.31) years. The mean and 95% confidence intervals (given in brackets) of height and body weight were, respectively, 166.06 (163.87–168.26) cm and 67.35 (62.96–71.73) kg. The differences in these parameters were not statistically significant to what is presented in Table 1. Anthropometric measurements such as body weight (±0.1 kg) and height (±0.5 cm) were used to assess differences in the nutritional status of patients. The control group followed the average Pole’s diet [24].

### 2.4. Gluten-Free Diet Adherance

Each patient from the GFDG before starting a GFD received comprehensive information on GFDs in the form of a generally available brochure prepared by the Polish Association of People with CD and on a GFD, and kept a dietary self-report diary monitored by a qualified clinical dietitian. A GFD was defined as the consumption of gluten-free natural and processed products containing ≤20 mg of gluten per 1 kg. Compliance with the GFD was verified by a qualified clinical dietitian and a test assessing familiarity with products that either contain or do not contain gluten. All participants received a sample GFD menu. Follow-up appointments with the clinical dietitian included analyses of the patients’ food diaries, the energy values of their diets and the distribution of macronutrients. They also involved education on proper distribution of meals and energy during the day, low glycemic index foods, reduced supply of simple carbohydrates, increased consumption of fiber, proper structure of fatty acid consumption, increased supply of omega-3 polyunsaturated fatty acids and reduced consumption of saturated and trans unsaturated fatty acids. All participants remained under constant endocrinological and clinical dietitian care at the Endocrinology Clinic. The adherence to the GFD was analyzed by using the adherence questionnaire [25]. In the GFDG, no poor or very poor GFD adherence was observed. According to questionnaire in the GFDG (*n* = 31), adherence to the GFD was: excellent (*n* = 9), good (*n* = 17) and fair (*n* = 5) [25].

### 2.5. Laboratory Tests

Serum concentrations of TSH, fT3, fT4, anti-TPO and anti-TG2 were determined in all patients at baseline (A1), after 3 (A2), 6 (A3) and 12 months (A4) of observation, using the electrochemiluminescent method (ECLIA) on the Roche Cobas model 6000 module 601 apparatus.

### 2.6. Statistical Analysis

Statistical analyses were performed with the use of Statistica 13.3 (StatSoft Inc., Cracow, Poland). The Shapiro–Wilk test was used to assess the normality of the analyzed parameters. Due to non-normal distribution, the Mann–Whitney non-parametric U test was used to assess the differences for medians. Statistical significance was set at *p* < 0.05. Moreover, 95% confidence intervals for medians were calculated.

The obtained values indicated a strong variability of the tested parameters. Therefore, an attempt was made to match the distribution of variables to the normal distribution by using logarithmic transformation. The Shapiro–Wilk test was used again to assess the normality of the transformed data. Yet, again, not all parameters followed a normal distribution, so their means were compared with the data obtained during the first appointment, using the non-parametric Wilcoxon signed-rank test. Statistical significance was set at *p* < 0.05. The analyses were performed separately for the control group and the dietary group. Additionally, standard error (SE) values were calculated.

## 3. Results

Patients’ mean age and BMI measured during the first appointment was 36.86 (mean) ± 8.64 (SD) years, and 25.31 (mean) ± 4.80 (SD) kg/m^2^, respectively. Baseline parameters in the entire sample (*n* = 62) varied greatly, as shown in Table 1.

No significant differences were found between the non-GFD (CG) and the GFD (GFDG) groups at baseline or after 3, 6, and 12 months of observation in terms of median concentrations of fT3, fT4, anti-TPO or anti-TG (Table 2). A significant reduction in TSH concentrations in the GFDG was demonstrated at endpoint (12 months). Although their median concentrations in the GFDG decreased at each follow-up, no significant differences were found between the GFDG and the CG in terms of anti-TPO and anti-TG (Table 2).

In the next stage of the study, changes in median concentrations of TSH, fT3, fT4, anti-TPO and anti-TG were analyzed in both groups (GC and GFDG), showing no evidence of any significant differences after 3 and 6 months of observation. After 12 months, however, significant differences were observed in the median concentrations of fT4 and TSH (Table 3).

After logarithmic transformation, separate analyses for the CG and the GFDG showed a significant decrease in anti-TPO concentrations after 3 months and anti-TG concentrations after 3, 6 and 12 months of GFD and a significant reduction in anti-TG concentrations at appointment 3 in the controls (Table 4). In GFDG, a reduction in TSH concentrations was observed after 3, 6 and 12 months, and an increase in fT4 concentrations after 6 and 12 months (Table 4).

## 4. Discussion

A GFD is the only treatment in celiac disease and other gluten-related disorders; however, some of the studies show that it can be favorable in autoimmune diseases, but available evidence concerning the use of a GFD in cAITD still remains unclear. In the present study, we have tried to evaluate if the GFD administration in HT can lead to improvement of immunological and hormonal parameters.

In our study, we showed that a GFD in cAITD patients without CD can lead to TSH level reduction and can increase fT4 level in comparison to the control group, which can indicate that a GFD improves intestinal absorption of levothyroxine. What is fully understandable in children with CD and AITD, as reported by Valentino et al., is where a GFD promotes levothyroxine dosage reduction and hypothyroidism improvement [26], but this is not clear in patients without gluten-related disorders. For example, Krysiak et al. did not observe any influence on TSH and fT3 concentrations in HT patients—even the anti-TG2 antibodies were elevated [27]. 

Initially, we did not observe any differences between the GFDG and the CG in anti-TPO and anti-TG antibodies concentrations; only after using the logarithmic transformation our results showed, in the GFDG, a decrease in anti-TPO levels after 3 months and in anti-TG levels after 3, 6 and 12 months, which can be confirmed by isolated reports indicating that a GFD may be beneficial in cAITD. It should be mentioned that in our study, the logarithmic transformation also showed the level of anti-TPO antibodies decrease after 6 months in the control group. According to Krysiak et al., there were some important differences in both studies: 6 months of a GFD in women with cAITD can reduce concentrations of anti-TPO and anti-TG antibodies [27]. The study of Krysiak et al. included 32 women with cAITD, freely assigned to the study and control groups, not treated with levothyroxine, with elevated serum levels of anti-tTG antibodies and without duodenal biopsy or CD [27]. In our study, all women were randomized into two groups using a randomization program; all of the 62 enrolled women were receiving levothyroxine and those with serum anti-tTG antibodies underwent gastroscopy to rule out CD.

Based on a 2-year follow-up of 90 patients with CD, Ventura et al. demonstrated that 2 years of GFD use led to the normalization of anti-TPO concentrations in 11 out of 13 anti-TPO positive patients with CD [28]. In any of our 62 patients during the 12-month follow-up, we did not observe a normalization of antithyroid antibodies, but perhaps a much longer time of observation is needed. A retrospective study by Cosnes et al. showed that the use of GFDs in patients with CD may secure against the initiation of the autoimmune process and be associated with a twice-lower risk of developing another autoimmune disease [29]. In our 12-month follow-up with any of the participating women from the GFDG and the CG we did not observe a development of clinical visible signs of additional autoimmune disease. 

However, numerous studies conducted mainly among people with CD fail to provide evidence of associations between a GFD and the course of cAITD. According to Mainardi et al., changing eating habits and switching to a GFD failed to reduce the concentrations of anti-thyroid antibodies in patients with cAITD and CD [30]. Likewise, a long-term observation of children from Sardinia with CD by Meloni et al. shows that a GFD does not protect against AITD [31]. In another study, Ansaldi et al. did not observe a significant difference in the incidence of AITD in children with CD regardless of their use of GFDs [32]. Similar results were reported by Diamanti et al. [33]. In a retrospective, nearly 14-year observation conducted among children, Cassio et al. showed an increase in the incidence of AITD from 12% to 24%, despite the use of GFDs [34]. According to Metso et al., the use of GFDs in patients with CD does not prevent the progression of the autoimmune process in HT [35].

The above-described changes in antibody concentrations observed in our and other studies can be explained by fluctuations in anti-TPO and anti-TG antibodies concentrations depending on factors such as gender and age, but they may also be seasonal [36]. Regardless of sex, anti-TG antibodies tend to be higher in summer and autumn and lower in spring and winter, whereas the concentrations of anti-TPO are higher in summer and lower in winter [36].

Large population studies on patients with CD do not confirm the beneficial effect of GFDs on the autoimmune process in HT, and so caution is advised when prescribing a GFD to patients with cAITD [37]. It seems that it is not so much the GFD itself as general diet modification (including nutrition education and healthy dietary habits promotion, such as increased fiber consumption) that may play an important role in the GFDG, especially in terms of improving the absorption of levothyroxine from the gastrointestinal tract.

Selenium deficiency, an element that reduces oxidative stress, may affect the function of regulatory T lymphocytes in cAITD, as well as contribute to reduced concentrations of anti-thyroid antibodies and improved thyroid function [38]. Available reviews and meta-analyzes of different publications show that the use of routine selenium supplementation in AITD raises many doubts and seems to be justified only in the case of confirmed selenium deficiency [39,40]. Our study group consisted of Polish women, and it is worth noting that in the average Pole’s diet, the deficiencies of iodine and selenium are a common occurrence [24].

It is worth emphasizing that in the GFDG, inducing a GFD was connected with a healthy lifestyle promotion and education by our expert dietitian. In this way, we wanted to protect our patients from potentially harmful GFD influences. It is of note that processed gluten-free products may be particularly rich in saturated fat and sodium, and low in protein and fiber [41]. Calvo-Lerma et al. emphasize that gluten-free products are not equivalent to gluten-containing products, and should be redesigned by using a healthy oil, pseudocereals and whole flour [42]. The unsupervised use of GFDs may be associated with a significant increase in the consumption of rice- or maize-based products. Rice can contain heavy metals such as arsenic, copper, cadmium and lead [43]. Maize and its products may contain mycotoxins (fumonisins), which are hepatotoxic, nephrotoxic, hepatocarcinogenic and cytotoxic [44]. Our expert dietitian has deeply discussed this topic with patients following a GFD.

Imbalanced GFDs can be connected with dysbiosis [45], which could be harmful for our patients and decrease intestinal absorption. Research on the composition of gut microflora in people on a GFD suggests its reduced biodiversity and reduced concentrations of short-chain fatty acids such as acetic and propionic butyric acid [46]. 

Although we wanted to design the study properly, we did not avoid some limitations. First of all, our study involved only women with HS. Needless to say, the levothyroxine therapy itself, administered in all of our patients due to hypothyroidism or subclinical hypothyroidism, could also affect anti-thyroid antibody levels [47,48].

Secondly, even though the patients were under the control of qualified clinical dietitian, who professionally evaluated the adherence to the GFD, there was always a risk of patients’ noncompliance, which could be eliminated only by measuring gluten peptides in urine or feces [49]. We assumed that control group was following the average Pole‘s diet, but it would be more precise to monitor this group of patients.

On top of that, we had to take into account the so-called non-celiac gluten sensitivity (NCGS), diagnosed based on the presence of clinical symptoms and their withdrawal in response to the elimination of gluten from the diet [50,51]. In such cases, a GFD could improve the absorption of levothyroxine in the small intestine, thus resulting in faster clinical euthyroidism at a lower dose [51].

## 5. Conclusions

Patients with cAITD should be tested for other autoimmune diseases, including CD. After excluding CD, a GFD may be attempted, but still there is no clear evidence that it should be recommended to every patient with cAITD. Further research is needed to evaluate the effect of nutrition on autoimmune diseases (AD), including cAITD. To conclude, in light of current knowledge, there are no clear indications to routinely follow a gluten-free diet because of Hashimoto’s thyroiditis, and it is necessary to perform more studies to assess if cAITD patients achieve the benefits of following a GFD. 

## Figures and Tables

**Table 1:** Characteristics of the study group (*n* = 62).

Parameter and Norm	Whole Group (*n* = 62)		CG (*n* = 31)		GFDG (*n* = 31)	
	Mean	Median	Mean	Median	Mean	Median
Age (years)	36.86 (34.72–39.00)	38.40 (32.90–41.10)	37.07 (33.83–40.31)	38.10 (32.30–42.50)	36.64 (33.66–39.63)	38.40 (32.80–42.70)
Height (cm)	165.92 (164.47–167.37)	165.50 (164.00–169.00)	166.06 (163.87–168.26)	168.00 (164.00–170.00)	165.77 (163.75–167.79)	165.00 (164.00–169.00)
Body weight (kg)	69.57 (66.43–72.70)	69.05 (62.40–73.70)	67.35 (62.96–71.73)	68.30 (59.20–76.20)	71.79 (67.19–76.38)	71.40 (62.70–79.80)
BMI (kg/m^2^)	25.31 (26.47–24.15)	24.77 (22.96–27.21)	24.53 (22.79–26.27)	24.80 (21.37–28.16)	26.27 (24.59–27.95)	24.74 (22.96–28.27)
TSH (0.270–4.200 µIU/mL)	2.83 (2.28–3.37)	2.38 (2.21–2.84)	2.41 (1.82–2.99)	2.31 (1.61–3.09)	3.25 (2.31–4.18)	2.44 (2.21–3.29)
fT3 (2.00–4.40 pg/mL)	2.95 (2.83–3.06)	2.85 (2.74–3.03)	3.03 (2.86–3.21)	2.95 (2.74–3.21)	2.86 (2.71–3.01)	2.80 (2.63–3.09)
fT4 (0.93–1.70 ng/dL)	1.30 (1.25–1.35)	1.30 (1.26–1.37)	1.34 (1.26–1.41)	1.29 (1.26–1.33)	1.27 (1.19–1.34)	1.27 (1.23–1.39)
anti–TPO (0–34 IU/mL)	207.61 (153.23–261.99)	140.25 (100.70–230.70)	153.63 (107.08–200.19)	114.40 (75.90–189.70)	261.59 (163.78–359.40)	207.30 (100.70–325.70)
anti–TG (0–115 IU/mL)	203.15 (155.47–250.84)	141.25 (83.98–254.70)	215.23 (146.67–283.97)	231.60 (105.20–310.60)	191.08 (121.03–261.12)	145.80 (95.80–259.32)

CG—control group; GFDG—gluten free diet group; the 95% confidence intervals are given in brackets. The means and medians for all parameters were not statistically significantly different (*p* > 0.05).

**Table 2:** Median concentrations of analyzed biochemical parameters throughout the observation period (*n* = 62).

Parameter and Norm	Observation Time	CG (*n* = 31)	GFDG (*n* = 31)	*p*
TSH (0.270–4.200 µIU/mL)	first visit	2.31 (1.61–3.09)	2.44 (2.21–3.29)	0.317
	3 months	2.26 (1.52–2.90)	1.67 (1.33–2.66)	0.437
	6 months	2.26 (1.40–3.48)	2.07 (1.51–2.64)	0.670
	12 months	1.93 (1.40–3.01)	1.44 (1.14–1.88)	**0.044**
fT3 (2.00–4.40 pg/mL)	first visit	2.95 (2.74–3.21)	2.80 (2.63–3.09)	0.211
	3 months	2.96 (2.78–3.28)	2.84 (2.60–2.98)	0.189
	6 months	2.84 (2.59–3.07)	2.76 (2.57–2.96)	0.244
	12 months	2.84 (2.71–2.98)	2.69 (2.51–3.03)	0.286
fT4 (0.93–1.70 ng/dL)	first visit	1.29 (1.26–1.33)	1.27 (1.23–1.39)	0.841
	3 months	1.29 (1.20–1.41)	1.32 (1.22–1.40)	0.813
	6 months	1.27 (1.17–1.47)	1.37 (1.24–1.47)	0.085
	12 months	1.32 (1.24–1.48)	1.42 (1.35–1.51)	0.177
anti–TPO (0–34 IU/mL)	first visit	114.40 (75.90–189.70)	207.30 (100.70–325.70)	0.079
	3 months	122.80 (54.68–248.70)	174.10 (94.81–303.50)	0.303
	6 months	155.00 (98.28–245.10)	141.85 (96.38–277.80)	0.662
	12 months	134.50 (97.54–212.70)	133.40 (94.79–301.00)	0.455
anti–TG (0–115 IU/mL)	first visit	231.60 (105.20–310.60)	145.80 (95.80–259.32)	0.447
	3 months	203.90 (55.61–352.19)	131.70 (44.75–299.15)	0.685
	6 months	210.30 (64.80–355.81)	100.13 (36.95–295.90)	0.317
	12 months	123.60 (51.40–330.20)	93.89 (41.50–327.25)	0.515

CG—control group; GFDG—gluten free diet group; the 95% confidence intervals are given in brackets; first visit—baseline appointment, 3 months—second visit, 6 months—third visit, 12 months—last visit; in bold font—statistically significant difference.

**Table 3:** Change in median concentrations (Δ) of analyzed parameters between individual appointments (*n* = 62).

Parameter and Norm	Observation Time	CG (*n* = 31)	GFDG (*n* = 31)	*p*
TSH (0.270–4.200 µIU/mL)	3 months	0.43 (−0.22/0.95)	0.89 (0.11/1.52)	0.091
	6 months	0.00 (−0.72/0.73)	0.77 (0.01/1.42)	0.114
	12 months	0.33 (−0.27/0.95)	1.17 (0.47/1.76)	**0.039**
fT3 (2.00–4.40 pg/mL)	3 months	0.15 (−0.06/0.37)	−0.03 (−0.20/0.17)	0.385
	6 months	0.23 (−0.04/0.49)	0.13 (−0.10/0.40)	0.921
	12 months	0.26 (−0.03/0.59)	0.12 (−0.08/0.33)	0.309
fT4 (0.93–1.70 ng/dL)	3 months	−0.03 (−0.10/0.11)	−0.04 (−0.7/0.14)	0.670
	6 months	0.01 (−0.07/0.11)	−0.08 (−0.17/0.01)	0.052
	12 months	−0.01 (−0.07/0.09)	−0.10 (−0.21/0.01)	**0.022**
anti–TPO (0–34 IU/mL)	3 months	3.98 (−11.85/20.82)	10.51 (−1.33/24.52)	0.119
	6 months	6.26 (−10.93/19.45)	5.89 (−13.54/28.31)	0.746
	12 months	−0.15 (−26.51/27.54)	4.40 (−37.63/51.87)	0.502
anti–TG (0–115 IU/mL)	3 months	2.67 (−12.35/32.68)	18.12 (7.78/44.21)	0.257
	6 months	7.85 (−6.23/59.31)	25.28 (−6.08/41.66)	0.472
	12 months	−1.96 (−62.54/42.69)	2.90 (−12.24/60.33)	0.182

The 95% confidence intervals are given in brackets; first visit—baseline appointment, 3 months—second visit, 6 months—third visit, 12 months—last visit; in bold font—statistically significant difference; positive Δ—decrease; negative Δ—increase.

**Table 4:** Change in concentrations of the tested parameters in CG and GDFG after logarithmic transformation.

Statistical Measure	CG (*n* = 31)				GFDG (*n* = 31)			
	First Visit	3 Months	6 Months	12 Months	First Visit	3 Months	6 Months	12 Months
	anti-TPO (IU/mL)							
Mean	4.73	4.66	4.87	4.77	4.91	4.77	4.81	4.75
SE	0.15	0.18	0.16	0.15	0.17	0.18	0.19	0.19
*p*	-	0.665	0.981	0.704	-	**0.010**	0.581	0.302
	anti-TG (IU/mL)							
Mean	4.80	4.67	4.65	4.73	4.71	4.62	4.60	4.48
SE	0.23	0.23	0.25	0.25	0.21	0.21	0.22	0.25
*p*	-	0.084	**0.016**	0.572	-	**0.023**	**0.019**	**0.049**
	TSH (µIU/mL)							
Mean	0.68	0.47	0.78	0.50	0.94	0.40	0.55	0.24
SE	0.12	0.18	0.18	0.21	0.12	0.18	0.14	0.19
*p*	-	0.285	0.737	0.222	-	**0.001**	**0.048**	**0.000**
	fT3 (pg/mL)							
Mean	1.07	1.04	1.02	1.03	1.04	1.04	0.99	1.01
SE	0.03	0.02	0.03	0.02	0.03	0.02	0.02	0.03
*p*	-	0.067	0.063	0.054	-	0.784	0.065	0.209
	fT4 (ng/dL)							
Mean	0.28	0.24	0.24	0.28	0.22	0.24	0.31	0.34
SE	0.03	0.03	0.04	0.03	0.03	0.03	0.02	0.03
*p*	-	0.509	0.442	0.665	-	0.290	**0.007**	**0.000**

CG—control group, GFDG—gluten free diet group; SE—standard error; first visit—baseline appointment, 3 months—second visit, 6 months—third visit, 12 months—last visit; in bold font—statistically significant difference.

## Data Availability

The data presented in this study are available on request from the corresponding author.
